# Concurrent crack and powder cocaine users from Sao Paulo: Do they represent a different group?

**DOI:** 10.1186/1471-2458-6-10

**Published:** 2006-01-20

**Authors:** Camila Guindalini, Homero Vallada, Gerome Breen, Ronaldo Laranjeira

**Affiliations:** 1 Department of Psychiatry, University of São Paulo Medical School, Brazil; 2 MRC Social Genetic and Developmental Psychiatry Research Centre, Institute of Psychiatry, King's College London, UK; 3 UNIAD (Unit of Drug and Alcohol Research), Department of Psychiatry, Federal University of São Paulo, Brazil

## Abstract

**Background:**

Cocaine abuse is a serious and socially damaging illegal drug problem. Different routes of administration are associated with a specific progression of use, different degrees of abuse liability, propensity for dependence and treatment response. There have been relatively few studies comparing different cocaine users groups and no studies into the characterization of the group of individuals reporting concurrent use of powder cocaine and crack cocaine.

**Methods:**

Six hundred and ninety-nine cocaine users were assessed during the period August 1997 to October 1998 in one outpatient and six inpatient clinics located in the São Paulo, Brazil. Patients were interviewed using a structured questionnaire schedule in Portuguese, designed specifically for the Brazilian population. The statistical analyses were performed using either ANOVA or a chi-squared test and focusing on their preferred form of use/route of administration and other variables.

**Results:**

For 83% of the variables tested in this study, the *Dual Users *subgroup (using both powder and crack cocaine) demonstrated statistical differences from the single drug user subgroups. Those differences include the initiation of cocaine, the abuse of other illicit drugs, and rates of criminal history.

**Conclusion:**

These data suggest cocaine-dependent individuals who report use of both powder and crack cocaine are an at least partially, distinct subgroup. However, further studies will be necessary to confirm this and to determine if they also show a different treatment response.

## Background

Cocaine abuse is one of the most serious and socially damaging illegal drug problems. The 2003 US National Survey on Drug Use and Health [1] found that 14.7% of the population of the United States aged 12 and older had tried cocaine at least once in their lifetimes. In Europe, the highest rate with a lifetime prevalence of 5.2% was reported for the United Kingdom [2]. In Brazil it is estimated that 10% of those aged 18–28 years in Sao Paulo have used cocaine at least once [3]. However, it remains unclear how many current users there are, or how many are prone to dependence.

The phases of dependence can be defined by different clinical characteristics in relation to dose, administration route, and length and pattern of cocaine use. There are positive correlations between drug abuse liability, intensity of intoxication and reported euphoria, with bioavailability of the substance in the central nervous system, and speed at which the maximum concentration in the brain is reached after ingestion. Consequently, route of administration can have important implications in the development of addictive behaviour(s). Crack cocaine, in comparison to cocaine hydrochloride, or powder cocaine, provides an immediate euphoric effect and is associated with high dependence liability and distinct treatment outcomes [4-6]. Intriguingly, studies investigating changes in routes of administration and patterns of cocaine use in Brazil demonstrated an important increase in the number of people reporting crack cocaine use in the last decade [7-12]. Although some of these studies identified three distinct groups of users [9,10,12]: 1) individuals who were only snorting cocaine, 2) individuals who were only smoking cocaine and 3) a large group of individuals who were using both routes of administration concurrently, characterization and comparisons have tended to consider two groups only: crack and powder cocaine users, where patients reporting more than one route were counted per route [9,12].

The present study describes the socio-demographic characteristics and drug abuse history among cocaine users from Sao Paulo, aiming to better characterize the different subgroups of users divided by their preferred form of use and route of administration, focusing on the analysis of dual users separately from the ones who use crack or powder cocaine only.

## Methods

### Sampling

Although this sample is essentially a convenience sample, it was intentionally selected from a wide range of services with diverse characteristics to avoid an ascertainment bias [13]. The study group consisted of drug users who were in detoxification, abstinence or counselling treatment from August 1997 to October 1998 in six inpatients and one outpatient clinic located in the metropolitan region of São Paulo City. The catchment area comprises the population of Sao Paulo and 39 smaller surrounding cities, the largest Brazilian (and South American) urban area. These drug treatment centres accounted for an estimated 75% of all hospitalizations related to drug abuse or illegal drug dependence carried out during the study period.

### Subjects

Inclusion criteria were: age 18 years and older, a history of primary or only cocaine abuse and under treatment at the selected centres. Individuals with history of exclusive alcohol dependence and mental disability were excluded. A total of 699 patients were interviewed and gave written informed consent. Subjects also gave a small sample of blood for genetic studies and HIV testing. This project was approved by the Ethical Committee of the Federal University of São Paulo and other relevant local ethics committees. HIV incidence investigation and a subgroup profile description were published previously [12,15].

### Measurements

A structure interview schedule in Portuguese had been designed specifically for the Brazilian population [14] and was comprehensively piloted previously. The questionnaire covers socio-demographic characteristics, sexual behaviours, and drug use profile. Responses to questions were dichotomous, multiple-choice or numeric in format. As the majority of the questions had a screening approach, the time taken to complete the interview was approximately 20 minutes (for details, see Ferreira-Filho et al, 2003). Interviews were conducted by three researchers, either experienced psychiatrists or psychologists who had been trained to use the questionnaire. All subjects satisfied an ICD10 diagnosis [16] of cocaine dependence (ICD 10 code F14.2).

### Statistical analyses

A datafile was created for use with SPSS statistical software, v.12.0. The statistical significance of differences between the groups was established using either an *ANOVA *test for comparisons of means or a χ^2 ^test for comparison of categorical data. In addition the *Kolmogorov-Smirnov Test *was used for analyses of continuous, non-parametric data.

## Results

The study sample comprised 699 Cocaine Users. The predominant preferred forms of administration were powder cocaine for 22.6% of the participants and crack cocaine for 7.9% while the majority, 69.5%, reported using both routes concurrently. In order to conduct a comparative analysis the sample was divided as follows: 1) *Powder Cocaine Users*, individuals who used cocaine or powder cocaine 2) *Crack Cocaine Users*, individuals who used the freebase processed cocaine hydrochloride commonly known as "crack" and 3) *Dual users*, individuals who reported using both forms of cocaine.

### Socio demographic characteristics

A summary of socio demographic data is presented in Table 1. Ninety-five per cent of the sample (n = 668) were male, and this proportion was consistent among the three cocaine groups. The same consistency between groups was found when marriage status was analyzed: 60.4% were single, 26% were married or co-habiting and 13.6% were divorced, separated or widowed at the time of the interview. However, when age (F = 4.3; df_b _= 2; df_w _= 696; p = 0.01), ethnic background (χ^2 ^= 7.9; df = 2; p = 0.01) and level of education (χ^2 ^= 14.1, df = 2, p = 0.001) were evaluated, statistic differences among groups could be identified.

*Dual Users *tended to be younger (26.2; SD = 6.8) and have a higher percentage (30%) of individuals with self-reported African ancestry than *Powder Cocaine Users *(28.1 years; SD = 7.7 and 20%, respectively); (F = 8.6, df_b _= 1; df_w _= 642, p = 0.003 and χ^2 ^= 5.7, df = 1, p = 0.017, respectively), but did not statistically differ from *Crack Cocaine Users *(26.8 years; SD = 7.3 and 37%, respectively), (F = 0.4, df_b _= 1; df_w _= 539, p > 0.05 and χ^2 ^= 1.2, df = 1, p > 0.05, respectively). Education attainment differed in the *Dual Users*, with 27% of the individuals having attended the secondary school/university (ages 15 and over), versus 38% in *Powder Cocaine Users *(χ^2 ^= 7.1, df = 1, p = 0.007) and was higher when compared with 13% in the *Crack Cocaine Users *(χ^2 ^= 4.9, df = 1, p = 0.02).

### Licit drug Use

Eighty-four percent (n = 588) of the patients were smokers (Table 2). The percentage of smoking individuals was 88% in the *Dual Users *versus 74% in *Powder Cocaine Users *(χ^2 ^= 16.6, df = 1, p < 0.0001) and 85% in *Crack Cocaine Users *(χ^2 ^= 0.2, df = 1, p > 0.05). Regarding alcohol consumption, *Dual Users *reported to drink more alcohol per week than *Crack Cocaine Users *(ks = 1.6, p = 0.01) Moreover 37% of the *Dual Users *reported consuming more than 50 units of alcohol per week, compared to 27% of the *Crack Cocaine Users *(χ^2 ^= 1.9, df = 1, p > 0.05) and 42% of the *Powder Cocaine Users *(χ^2 ^= 3.6, df = 1, p = 0.05).

### Illicit drug use

When history of other illegal drugs was evaluated in the three groups, differences were found for cannabis (χ^2 ^= 18.6, df = 2, p < 0.0001) and solvents (χ^2 ^= 35.2, df = 2, p < 0.0001) but not for heroin use (χ^2 ^= 2.6, df = 2, p > 0.05, see Table 2). The proportion of individuals that had ever used cannabis was higher in the *Dual Users *group when compared to both *Powder Cocaine Users *group (χ^2 ^= 11.4, df = 1, p = 0.001) and *Crack Cocaine Users *(χ^2 ^= 16.4, df = 1, p < 0.001). Similar figures were observed for the use of solvents, with a increased number of individuals in the *Dual Users *group reporting to have ever used this substance in their life, compared to the *Powder Cocaine Users *and *Crack Cocaine Users *groups (χ^2 ^= 22.9, df = 1, p < 0.0001 and χ^2 ^= 20.1, df = 1, p < 0.0001, respectively).

The use of both solvents and heroin in the last month prior to admission was low overall and did not differ among groups. The highest rates were 1.8% for the use of solvents by the *Crack Cocaine Users *group and 0.8% for use of heroin in the *Dual Users *group. While the reported use of cannabis in the last month was high in all groups, it was particularly raised in the *Dual Users *(63%) as compared to *Powder Cocaine Users *(43%, χ^2 ^= 19.2, df = 1, p < 0.001) and *Crack Cocaine Users *(45%, χ^2 ^= 6.3, df = 1, p = 0.01).

### Initiation into cocaine use

**Figure 1:**
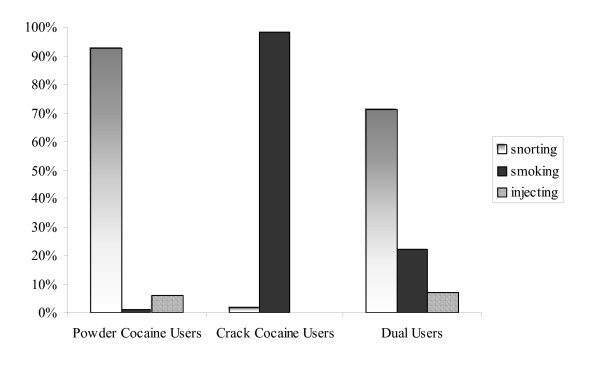
The first route of administration in the *Powder Cocaine Users*, *Crack Cocaine Users *and *Dual Users *groups.

The most common first routes of cocaine administration were snorting (n = 571), then smoking (n = 184) and injecting (n = 50) – counting per route for individuals who reported two or more concomitant first routes. Separating the groups, snorting was the first route of choice for 93% of the *Powder Cocaine Users *and for 71% of the *Dual Users*. However, in the *Crack Cocaine Users *group, the first chosen route was smoking in 92% of the individuals (Figure 1), followed by snorting cocaine in only 8% of the individuals. The mean age of initiation of cocaine use was significantly different among the groups. *Dual Users *tend to start using cocaine earlier (18.3 years, SD = 5.4) than the *Powder Cocaine Users *(20.7 years, SD = 7.4) (F = 18.1, df_b _= 1; df_w _= 641, p < 0.0001) and the *Crack Cocaine Users*. (22.7 years, SD = 8.1) (F = 28.4, df_b _= 1; df_w _= 539, p < 0.0001).

### Criminal history

In relation to criminal history, 53% of the patients had been in prison at some stage in their life. This rate was higher in the *Dual Users *group (59%) in comparison to the *Powder Cocaine Users *group (36.7%) (χ^2 ^= 23.7, df = 1, p < 0.0001) and was non-significantly lower in the *Crack Cocaine Users *(49%) (χ^2 ^= 2.6, df = 1, p = 0.1).

## Discussion

In this study we describe socio-demographic characteristics and history of licit and illicit drugs of abuse among 699 Cocaine Users recruited from seven drug treatment hospitals located in the metropolitan region of Sao Paulo, Brazil. The analyses were conducted dividing the sample according to the preferred form of cocaine administration: 1) *Powder Cocaine Users*; 2) *Crack Cocaine Users *and 3) *Dual users*. The high frequency of concomitant use of crack with powder cocaine in our sample may be partly due to an increased availability – and a concomitant reduction in the price – of crack cocaine that occurred in the early 1990's, which led to an explosion in consumption. Dunn *et al*. (1996) [7] and Ferri and Gossop (1999, 2001) [9,10] also reported increased crack use among patients who sought treatment in Sao Paulo clinics over this period. This had important social implications since crack cocaine users are more likely to report craving and impaired control of their drug use, tend to present higher levels of severity of dependence, higher involvement in prostitution, violence and crime [17,18].

With respect to socio-demographic characteristics, our sample was essentially comprised of young, single, men with mainly primary education, corroborating previous published data about cocaine users attending treatment in Sao Paulo [8,9,11]. Comparing the different groups, *Dual Users *tended to differ from *Powder Cocaine Users*, regarding age, self-reported ethnicity, educational attainment and smoking status. Moreover, a high proportion of alcohol consumption was found in our sample with *Dual Users *reporting to drink more alcohol per week than *Crack Cocaine Users*. High prevalence for concurrent alcohol and cocaine misuse has been reported [9,10]. Epidemiological studies indicate that simultaneous abuse of powder cocaine and alcohol is widespread [19] and it is known that concurrent ingestion of cocaine and alcohol results in cocaethylene production [20,21]. Cocaethylene is a pharmacologically active homolog of cocaine with an increased metabolic half-life and may play a role in reinforcement effects of cocaine.

In addition, regarding the history of use of illicit drugs, *Dual Users *demonstrated a higher proportion of individuals that had ever used cannabis and solvents in their life and cannabis in the last month. Polydrug abuse is a phenomenon reported by cocaine users in several other studies [7,10,22] and represents an important concern to treatment and public health strategies. Polydrug users show complex life histories and are normally associated with poorer treatment outcomes than those using one drug only [22]. The latest data on hospital emergency visits estimates that more than half of the visits related to "drug misuse or abuse" involved more than one drug [23] and recent studies reported that simultaneous use of multiple drugs has been shown to contribute substantially to overdose mortality [24,25]. However, contrasting with the previously mentioned studies and with surveys in Europe where the consumption of opiates by cocaine users was important [26], the recent consumption of heroin was particularly low in our sample, reported by only 4 patients, less than 1% of the sample."

Eight-one per cent of our sample began using cocaine by snorting, agreeing with a previous study where 84% of the cocaine users in Sao Paulo [8] had chosen this route on the first occasion. Although the majority of the sample reported first use of cocaine by this route, 68% of those individuals went on to experience crack cocaine and 9% injected cocaine at some time of their life. However, when current *Crack Cocaine Users *were analyzed separately, ninety-two per cent of those reported first use of cocaine by smoking and, here, a smaller proportion of individuals went on experience powder cocaine (43%) or injected cocaine (4%). Interestingly most *Dual Users *(66%) started using powder cocaine and then moved onto crack, supporting the suggestion that hydrochloride used intranasally may be a gateway drug or behaviour to using crack cocaine [17].

The presence of substance abuse and dependence are particularly associated with an increased risk of violent/homicidal behaviours [27]. Criminal activity was notably present in our sample. The *Dual *users groups were more likely to have been imprisoned in their life than the *Powder Cocaine Users*. Hatsukami and Fischman (1996) [17] reported crack cocaine to be linked with crime to a greater extent than cocaine hydrochloride, with many of these crimes being associated with the maintenance of drug consumption.

In summary, we found a high consumption of crack cocaine among the patients, corroborating with a historic change in the preference of cocaine use in Brazil in the past decade, reported by earlier studies. Furthermore we showed for the first time that interesting differences exist between *Powdered*, *Crack *and *Dual Cocaine Users*, a separate group, usually not considered in previous studies. The findings suggest that the *Dual Users *group differs from the mono users in several of the variables tested and appeared to be a distinct and more severe group regarding history of illicit drug abuse and rates of criminal history. They tended to have consumed significantly more cannabis and solvents. Moreover they also started consuming cocaine earlier in life and they were more likely to be actively involved in crime, showing higher percentage of individuals who have been arrested at least once in their life.

## Conclusion

In conclusion, our results tended to agree with previous studies about cocaine and crack use in Brazil while offering some new information regarding the individuals who report use of both powder and crack cocaine. In the present study, this group presents differences to mono users, which suggests these individuals may be considered as a separate group. The main limitations of this study were that a small amount of information was available for certain key variables, e.g., for criminal history we did not record whether or not the conviction was for anti-social or violent behaviour, and that we had to rely on patient self report for other measures such as concomitant drug and alcohol use. However, the sample was large and from seven different centres, thus we might expect that the observations are clinically relevant, at least for the urban region of Sao Paulo.

## Competing interests

The author(s) declare that they have no competing interests.

## Authors' contributions

CG and GB analyzed the data, carried out statistical analysis and wrote the paper. RL was the psychiatrist coordinator of the sample collection. RL and HV participated in study design, and helped to revise drafts of the manuscript. All authors read and approved the final manuscript.

## Pre-publication history

The pre-publication history for this paper can be accessed here:



## Figures and Tables

**Table 1:** Comparison of socio-demographic characteristics among *Powder Cocaine Users*, *Crack Cocaine Users *and *Dual Users *groups.

	**Powder Cocaine Users**		**Crack Cocaine Users**		**Dual Users**		**Total**	**pvalue**
*Age*	28.1		26.8		26.2		26.7	p = 0.01
	SD = 7.7		SD = 7.3		SD = 6.8		7.2	
*Ethnicity*								
Caucasian	125	80%	34	63%	339	70%	498	p = 0.01
African	31	20%	20	37%	143	30%	194	
*Sex*								
Male	149	94%	54	98%	465	96%	668	p > 0.05
Female	9	6%	1	2%	21	4%	31	
*Education*								
primary school or less	92	62%	47	87%	347	73%	486	P = 0.001
secondary school or more	57	38%	7	13%	127	27%	191	
*Marriage Status*								
single	96	61%	34	62%	289	60%	419	p > 0.05
married/cohabiting	37	23%	15	27%	128	27%	180	
divorced/widowed	25	16%	6	11%	63	13%	94	

**Table 2:** Comparison of history of legal and illegal drugs in the *Powder Cocaine Users*, *Crack Cocaine Users *and *Dual Users *groups and in the total sample.

	**Powder Cocaine Users**		**Crack Cocaine Users**		**Dual Users**		**Total**	**pvalue**
*Alcohol*								
<50 units/week	92	58%	40	73%	299	63%	431	p > 0.05
>50 units/week	66	42%	15	27%	175	37%	256	
*Smoking*								
yes	117	74%	46	85%	425	88%	588	p > 0.001
no	41	26%	8	15%	60	12%	109	
*Cannabis*								
never used	11	7%	6	11%	8	2%	25	p > 0.0001
ever used	147	93%	49	89%	469	98%	665	
*Solvents*								
never used	70	44%	29	53%	118	24%	217	p > 0.0001
ever used	88	56%	26	47%	367	76%	481	
*Heroin*								
never used	153	97%	54	98%	459	94%	666	p > 0.05
ever used	5	3%	1	2%	27	6%	33	
